# La forme pseudo tumorale de la tuberculose primitive du nasopharynx: à propos de deux nouvelles observations et revue de la littérature

**DOI:** 10.11604/pamj.2013.14.63.1325

**Published:** 2013-02-14

**Authors:** Mohammed Touati, Abdelfettah Aljalil, Mehdi Chihani, Rachid Bouchentouf, Brahim Bouaity, Haddou Ammar

**Affiliations:** 1 Service ORL et CCF, Hôpital Militaire Avicenne de Marrakech, Maroc; 2 Service pneumologie, Hopital militaire Avicenne, Marrakech, Maroc

**Keywords:** Tuberculose, nasopharynx, diagnostic histologique, Tuberculosis, nasopharynx, histological diagnosis

## Abstract

La tuberculose primitive du nasopharynx est rare, nous présentons deux observations révélées par un aspect pseudo tumoral et à travers lesquelles nous soulevons le problème de diagnostic différentiel avec les lésions malignes du nasopharynx. La première observation concerne un jeune patient de 22 ans hospitalisé pour obstruction nasale bilatérale évoluant dans un contexte d'apyrexie et de conservation de l’état général. La nasofibrosopie et le scanner ont monté un processus tumoral évoquant une hypertrophie des végétations adénoïdes. Le deuxième cas est celui d'un homme de 45 ans tabagique chronique qui a présenté une adénopathie latérocervicale droite, une obstruction nasale et une otite séromuqueuse homolatérale. La nasophibroscopie et le scanner on montré un bourgeon tumoral postéro latéral droit du nasopharynx évoquant un carcinome nasopharyngé. Les biopsies du nasopharynx et les études histologiques, chez les deux patients, ont confirmé le diagnostic de tuberculose. La recherche d'autres localisations était négative. Le pronostic était favorable après 6 mois de traitement antibacillaire. La tuberculose primitive du cavum est rare, elle revêt le plus souvent des formes pseudotumorales et pose des problèmes de diagnostic différentiel avec les tumeurs nasopharyngées, son pronostic sous traitement antibacillaire est.

## Introduction

La tuberculose est l'une des maladies infectieuses les plus répandues dans le monde avec environ 9millions de nouveaux cas par an, causant 2 millions de décès, son incidence a augmenté depuis l'invasion de l’épidémie du SIDA, l'accroissement de la démographie, la pauvreté et la migration des populations [1]. Dans notre pays cette infection sévit de façon endémique, et peut prendre des formes pouvant faire errer le diagnostic. Les localisations au niveau de la sphère ORL sont souvent secondaires ou associées à des formes pulmonaires, les formes primitives bien que rares ne sont pas exceptionnelles [2]. Nous rapportons deux nouvelles observations de la tuberculose primitive du cavum d'aspect pseudo tumoral, à travers lesquelles nous rappelons les différents aspects et les difficultés diagnostiques de cette localisation.

## Patient et observation

### Cas 1

Patient âgé de 22 ans, sans antécédents pathologiques particuliers, hospitalisé pour bilan d'une obstruction nasale bilatérale avec rhinorrhée purulente, évoluant depuis trois mois dans un contexte d'apyrexie et de conservation de l’état général. L'examen clinique a montré une muqueuse nasale congestive, une rhinorrhée postérieure purulente, une otite séromuqueuse bilatérale avec une perte auditive moyenne de 25 dB, la palpation cervicale n'a pas retrouvé d'adénopathies. La nasofibroscopie a objectivé la présence d'un processus tumoral occupant la quasitotalité du rhinopharynx, de surface irrégulière, couvert de sécrétions mucopurulentes et obstruant les deux choanes.

**Figure 1:**
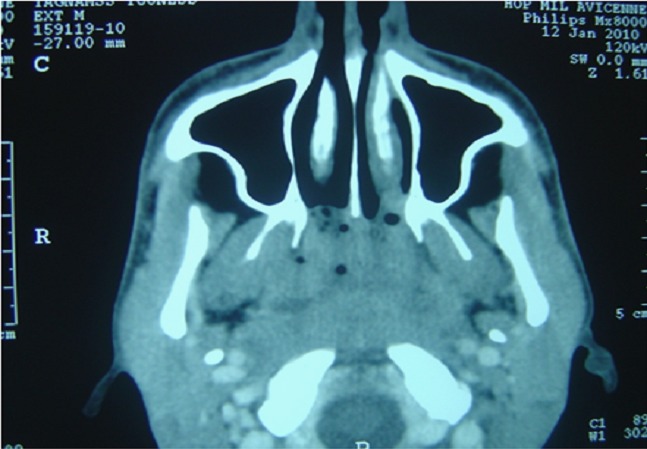
TDM du cavum (premier patient) en coupe axiale montrant un processus tumoral au dépend de toutes les parois du cavum, comblant les fossettes de Rosenmüller sans lyse osseuse avec des bulles d'air piégées au sein du processus.

**Figure 2:**
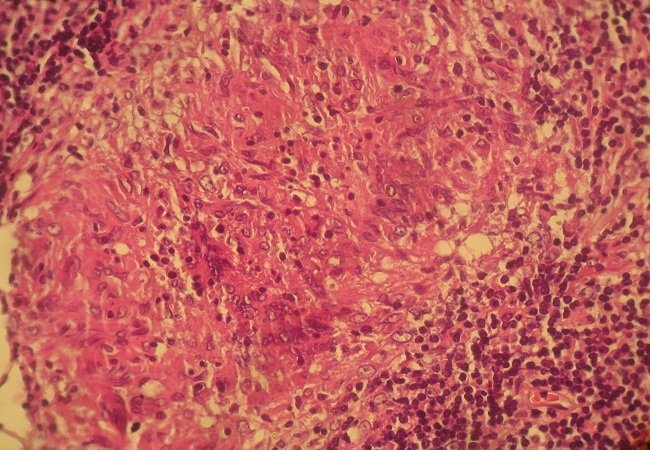
Coupe histologique montrant une muqueuse nasopharyngée siège d'un granulome épithélio-giganto-cellulaire avec nécrose caséeuse attestant une tuberculose nasopharyngée évolutive.

Le scanner cervical a montré un processus tumoral prenant toutes les parois du cavum, comblant les fossettes de Rosenmuller, étendu aux choanes, sans lyse osseuse, avec présence de bulles d'air piégées au sein du processus (Figure 1). La crainte d'un carcinome du cavum, nous a incité à faire une biopsie du cavum dont le résultat histologique était une surprise en montrant la présence d’ un granulome épithélio-giganto-cellulaire avec nécrose caséeuse (Figure 2), sans signes de malignité associés. Le bilan biologique a montré un syndrome inflammatoire modéré, l'IDR à la tuberculine était positive, la radiographie pulmonaire était normale.

### Cas 2

Patient âgé de 45 ans, tabagique chronique, admis dans le service pour exploration d'une adénopathie latérocervicale droite, apparue depuis 4 mois et augmentant progressivement de taille dans un contexte d'amaigrissement non chiffré, accompagnée d'obstruction nasale et d'hypoacousie homolatérales.

**Figure 3:**
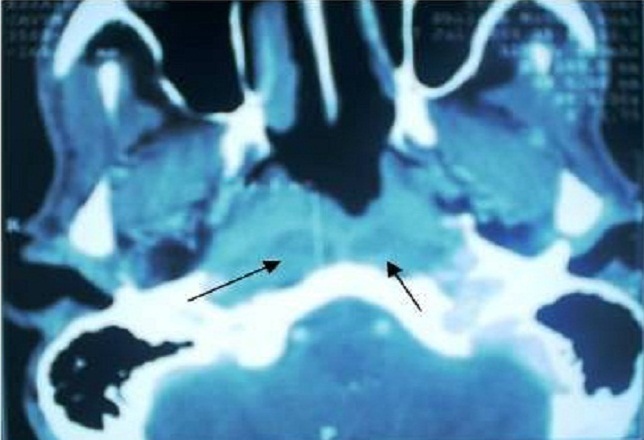
TDM du cavum (deuxième patient) en coupe axiale montrant un processus tumoral de la paroi postéro latérale droite du rhinopharynx avec adénopathies rétro pharyngées (flèches).

L'examen clinique a retrouvé une adénopathie sous digastrique droite, ferme, indolore, et mesurant 3cm de diamètre, une otite séromuqueuse droite. La nasofibroscopie a mis en évidence un bourgeon tumoral de la paroi postéro latérale droite du cavum. Le scanner cervical a montré un processus tumoral de la paroi postérieure du cavum avec respect de la graisse parapharyngée et présence d'adénopathies rétroparyngées (Figure 3).

Des biopsies multiples du cavum ont été réalisées et l’étude anatomopathologique a montré la présence de granulomes épithélio-giganto-cellulaires avec nécrose caséeuse. Le bilan biologique a montré une IDR positive et un syndrome inflammatoire manifeste, la radiographie pulmonaire était normale.

**Figure 4:**
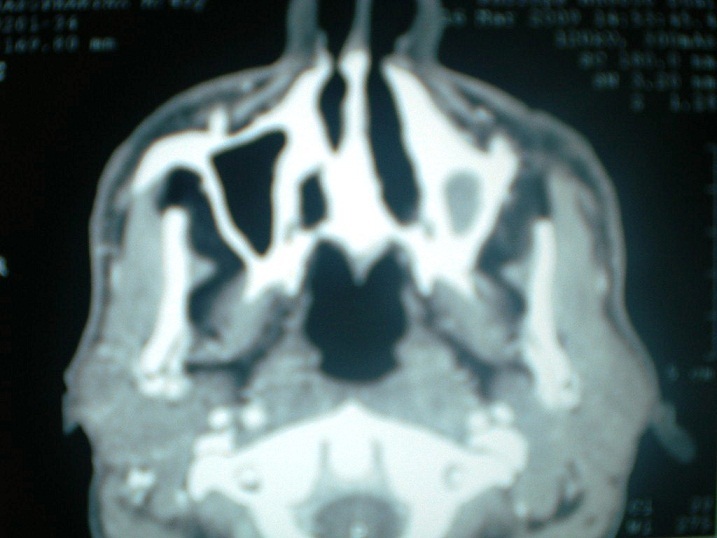
TDM de control (deuxième patient) montrant la régression totale des lésions tuberculeuses après traitement antibacilaire.

La recherche d'une autre localisation de la maladie chez nos deux patients, notamment pulmonaire, les bascilloscopies dans les crachats et le liquide de tubage gastrique et la sérologie HIV, étaient négatives, ceci nous a permis de retenir le diagnostic de tuberculose pseudo tumorale primitive à localisation rhinopharyngée chez les deux patients. Un traitement antibacillaire a été instauré, associant la rifampicine, l'isoniazide et le pyrazinamide pendant deux mois, suivi d'un relais de quatre mois de rifampicine et d'isoniazide. L’évolution sous traitement a été satisfaisante, le contrôle après trois mois d'arrêt du traitement a montré une régression des signes cliniques, endoscopiques et radiologiques (Figure 4). Des biopsies de contrôle avec études histologiques ont monté une stérilisation rhinopharyngée sans signes histologiques de malignité. Les patients sont toujours suivis de façon régulière sans aucune récidive locale avec un recul moyen de 18mois.

## Discussion

La tuberculose du nasopharynx correspond à l'ensemble des lésions évolutives de type granulomateux de la muqueuse consécutive à l'infection par le bacille de koch, c'est une localisation rare de la maladie dans sa forme primitive bien que le cavum soit richement vascularisé et situé dans une région très largement exposée, elle a été initialement décrite par GRAFF en 1936 [2].

Le mode de contamination cavaire suppose une inoculation locale par inhalation de poussières bacillifères, ou bien une dissémination par voie sanguine ou lymphatique à partir d'un foyer méconnu d'où l'aspect de localisation primitive, ou à partir d'un foyer connu surtout pulmonaire pour la voie hématogène ou bien muqueux pour la voie lymphatique [3, 4].

Sur la plan clinique, la tuberculose primitive du cavum a une expression peu spécifique, elle revêt le plus souvent des formes pseudotumorales. Tsé et Al ont publié en 2003 une série de 17cas de tuberculose du cavum, essentiellement des formes pseudotumorales [4]. Les signes cliniques sont semblables à ceux d'un carcinome nasopharyngé [4, 5], avec adénopathie cervicale généralement unilatérale, obstruction nasale homolatérale, une épistaxis, une rhinorrhée purulente sale avec jéttage postérieur ou bien parfois des signes otologiques à type d'hypoacousie secondaire à une otite séromuqueuse (cas de nos deux patients).

L'examen endoscopique du cavum est impératif, plusieurs aspects de la tuberculose cavaire ont été décrits dans la littérature: une ulcération, une tuméfaction irrégulière ulcérobourgeonnante, une hypertrophie muqueuse régulière ou même parois un aspect d'hypertrophie des végétations adénoïdes, et tous ces aspects pourrait très bien correspondre à une pathologie maligne [3, 6, 8].

Les signes radiologiques ne sont pas spécifiques et sont surtout en faveur d'un processus tumoral, le scanner et l'IRM permettent d'affirmer l'absence de caractère invasif de cette tumeur [7].

Le diagnostic positif de la tuberculose du cavum ne peut etre confirmé que par l'examen histologique avec présence de granulomes épithélio-giganto-cellulaires avec nécrose caséeuse, tous les signes cliniques endoscopiques et radiologiques sont surtout en faveur d'une étiologie tumorale, d'où la nécessité de réaliser plusieurs biopsies à endroits différents pour pouvoir éliminer un carcinome nasopharyngé ou bien une association qui reste possible mais exceptionnelle [3, 9], l'identification du germe par examen directe ou après culture sur milieu spécifique nécessite 4 à 6semaines et peut se révéler infructueuse, les techniques modernes comme la PCR permettent un diagnostic plus précoce et peuvent parfois trancher devant un granulome épithélio-gigonto-cellulaire sans nécrose caséeuse [10].

Le traitement de la tuberculose primitive du cavum est médical reposant sur une polychimiothérapie antibacillaire, l'association classique utilisée est une trithérapie à base de rifampicine, isoniazide et de pyrazinamide pendant 2 à 3 mois et suivie d'un relais pendent 4 à 6 mois par une bithérapie (isoniazide et rifampicine) [3, 10]. L'efficacité thérapeutique est basée sur la régression des signes cliniques et endoscopiques, toute évolution anormale doit faire évoquer une résistance ou bien une possibilité d'affection néoplasique coexistante, d'où la nécéssité de biopsies itératives [10]. Le risque de rechute est estimé à 1%, du essentiellement à l'apparition de souches de BK multirésistantes [2].

## Conclusion

Bien qu'il s'agisse d'une affection rare, la tuberculose primitive du cavum mérite d’être rappelée. La présence de nombreuses similitudes cliniques, endoscopiques et radiologiques avec les affections malignes du nasopharynx pose souvent un problème de diagnostic différentiel, son pronostic sous traitement antibacillaire est généralement bon, les échecs sont surtout liés à l’émergence de souches multirésistantes aux traitements.
